# Congenital Adrenal Hyperplasia in the Mediterranean: A Concise Overview

**DOI:** 10.3390/ph19050741

**Published:** 2026-05-08

**Authors:** Pavlos Fanis, Nicos Skordis, Marios Tomazou, Leonidas A. Phylactou, Vassos Neocleous

**Affiliations:** 1 Department of Molecular Genetics, Function and Therapy, The Cyprus Institute of Neurology and Genetics, Nicosia 1683, Cyprus; pavlosf@cing.ac.cy (P.F.); nicosskordis@paedi.org.cy (N.S.); vassosn@cing.ac.cy (V.N.); 2 School of Medicine, University of Nicosia, Nicosia 2417, Cyprus; 3 Division of Paediatric Endocrinology, Paedi Center for Specialized Paediatrics, Nicosia 2024, Cyprus; 4 Department of Bioinformatics, The Cyprus Institute of Neurology and Genetics, Nicosia 1683, Cyprus; mariost@cing.ac.cy

**Keywords:** Congenital Adrenal Hyperplasia, *CYP21A2*, 21-hydroxylase deficiency, newborn screening, pharmacogenomics, Mediterranean region, glucocorticoid therapy, epidemiology

## Abstract

**Background:** Congenital Adrenal Hyperplasia (CAH) is a group of autosomal recessive disorders caused by impaired adrenal steroidogenesis, most frequently due to pathogenic variants in the *CYP21A2* gene leading to 21-hydroxylase deficiency (21-OHD). Epidemiology and management vary across the Mediterranean Basin as a result of genetic and healthcare differences. **Objective:** To provide an overview of the epidemiology, diagnostic approaches and treatment patterns of CAH in Mediterranean countries. **Methods:** A structured review of the literature was performed using PubMed, using combined disease-related, geographic and methodological terms. Eligible studies reporting on epidemiology, diagnosis, or management of CAH were included. Data on study design, population characteristics, incidence, diagnostics, genetics and treatment availability were extracted. **Results:** Data were collected from 23 Mediterranean and neighboring regions covering over 8.7 million screened newborns. In countries with established newborn screening (e.g., Spain, Italy, France, Greece), the incidence of classic CAH ranged from 1:10,000 to 1:25,000 live births. Higher rates were reported in parts of North Africa and the Eastern Mediterranean. Diagnostic set-up and access to biochemical and genetic confirmation varied widely. Hydrocortisone remains the primary therapy, while access to mineralocorticoids and modified-release glucocorticoids differed across settings. **Conclusions:** Overall, considerable heterogeneity in CAH epidemiology and care exists across the Mediterranean region. Genetic factors such as founder effects, consanguinity and healthcare organization contribute to these differences. Expanding newborn screening, improving diagnostics and availability to treatments are critical to reducing disparities in CAH care.

## 1. Introduction

Congenital Adrenal Hyperplasia (CAH) refers to a group of autosomal recessive disorders primarily caused by defects in enzymes involved in the biosynthesis of glucocorticoids, mineralocorticoids and sex steroids from cholesterol in the adrenal cortex. The most common form, accounting for approximately 90–95% of cases, is due to mutations in the *CYP21A2* gene, which encodes the 21-hydroxylase enzyme (21-OH) [1,2,3]. Less commonly, CAH can result from mutations in other genes encoding steroidogenic enzymes, including: *CYP11B1* (11β-hydroxylase deficiency), *HSD3B2* (3β-hydroxysteroid dehydrogenase deficiency), *CYP17A1* (17α-hydroxylase/17,20-lyase deficiency), *STAR* (steroidogenic acute regulatory protein deficiency), *CYP11A1* (cholesterol side-chain cleavage enzyme deficiency) and *POR* (P450 oxidoreductase deficiency) [4]. These rarer forms of CAH also disrupt cortisol synthesis, leading to elevated adrenocorticotropic hormone (ACTH) levels and subsequent adrenal cortex hyperplasia [5].

Understanding the epidemiology of CAH is essential for identifying high-risk populations and optimizing strategies for early detection and treatment. These epidemiological patterns demonstrate significant variation across ethnic and geographic groups, shaped by factors such as genetic drift, consanguinity, founder effects and disparities in healthcare infrastructure [6]. The incidence of classic CAH, including salt-wasting (SW) and simple virilizing (SV) phenotypes, is estimated globally at approximately 1:10,000–20,000 live births [1,2,5,6,7,8,9,10,11,12]. In contrast, Non-Classic (NC)-CAH or late-onset CAH, is far more common with an incidence of ~1:1000 live births, rising to 1:100–200 in certain ethnic groups [5,10,11]. Among Ashkenazi Jews, carrier frequency is as high as 1 in 3, with reported prevalence up to 1 in 27 [11]. Other rates include 1:53 in Hispanics, 1:62 in Yugoslavs and 1:300 in Italians [13]. Nevertheless, NC-CAH is often underdiagnosed, as it usually manifests later in life with features such as premature adrenarche, hirsutism, menstrual irregularities or subfertility and is not detected by newborn screening, leading to an underestimation of its true prevalence [14,15,16,17].

The Mediterranean Basin, marked by high genetic diversity and centuries of population admixture [18,19], demonstrates notable variation in both the incidence of CAH and the availability of specialized care. Recent advances in pharmacogenomics have highlighted the role of *CYP21A2* pathogenic variants in influencing disease severity and individual responsiveness to glucocorticoid therapy. Integrating molecular diagnostics and pharmacogenomic profiling into routine clinical pathways is therefore crucial for optimizing treatment strategies and informing evidence-based health policy [20,21].

Despite the increasing literature on CAH epidemiology, major gaps exist regarding region-specific data, mainly within the Mediterranean. Existing studies are heterogeneous in diagnostic methodologies, population coverage and treatment approaches, limiting compatibility and understanding of disease burden across the region. Moreover, variation in newborn screening implementation and access to biochemical and genetic testing further complicates accurate estimation of incidence and prevalence across countries. To address these discrepancies, this concise overview is suitable for this purpose and aims to map evidence on the incidence, prevalence, genetic features and treatment practices of CAH in Mediterranean populations by (i) summarizing rates of classic and non-classic CAH; (ii) portraying the spectrum of *CYP21A2* pathogenic variants; and (iii) evaluating the influence of demographic and healthcare factors on detection and treatment patterns.

## 2. Methodology

### 2.1. Search Strategy

A structured literature search was conducted in PubMed (https://pubmed.ncbi.nlm.nih.gov, accessed on 10 March 2026) to identify studies on CAH in Mediterranean and neighboring countries. The search strategy was organized using three groups of terms, combined to ensure comprehensive coverage. The first group included disease-specific terms such as Congenital Adrenal Hyperplasia, 21-Hydroxylase deficiency, Classic CAH and NC-CAH. The second group comprised geographic terms referring to countries in the Mediterranean region, Italy, Spain, Greece, Cyprus, Turkey, Lebanon, Israel, Algeria, Tunisia, Morocco, Croatia, Southern France, Slovenia, Albania, Egypt, Libya and Syria. The third group included research-related terms such as epidemiology, founder effect and pharmacogenomics. In addition, to broaden the scope of relevant evidence, PubMed article Type Filters were applied, covering adaptive clinical trials, case reports, clinical studies and trials (Phase I/II, randomized, controlled, multicenter), observational studies, validation studies, meta-analyses, reviews, systematic reviews, scoping reviews, veterinary studies, classical articles and books/documents.

### 2.2. Data Extraction and Quality Assessment

Data extraction was performed independently by two reviewers using a standardized template. For each included study, data were systematically extracted on study characteristics (author, year of publication, country, study design and sample size). Population details included age, sex distribution and where available, genetic background. The clinical focus of each study was categorized according to the type of CAH (classic vs. non-classic) and the specific genetic subtype (e.g., 21-hydroxylase deficiency). Finally, outcomes of interest comprised prevalence or incidence rates, identification of founder mutations and pharmacogenomic findings. Disagreements during extraction were resolved by discussion and, if required, judgment by a third reviewer. The overall quality of evidence across the included studies was based in the synthesis, considering study design, sample size and consistency of findings. In addition, potential publication bias, underreporting and differences in case ascertainment related to the presence or absence of national newborn screening (NBS) programs were qualitatively assessed. Particular consideration was given to the possibility of underdiagnosis in regions relying mainly on clinical identification rather than systematic screening.

## 3. Results

### 3.1. Epidemiology, Diagnosis and Treatment Patterns of Congenital Adrenal Hyperplasia in the Mediterranean Basin

**Figure 1:**
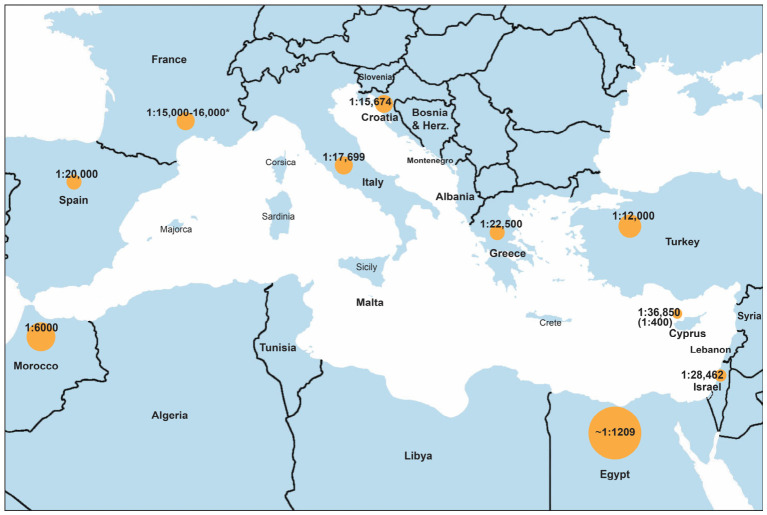
Geographic variation in classic CAH incidence across the Mediterranean region. Circle size reflects relative incidence, highlighting higher frequencies in North Africa and the Eastern Mediterranean compared to Southern Europe. The value in parentheses for Cyprus indicates the estimated carrier frequency for non-classic CAH (NC-CAH). * The value reported for France is derived from national newborn screening data for CAH, which include populations from the southern regions of the country.

**Figure 2:**
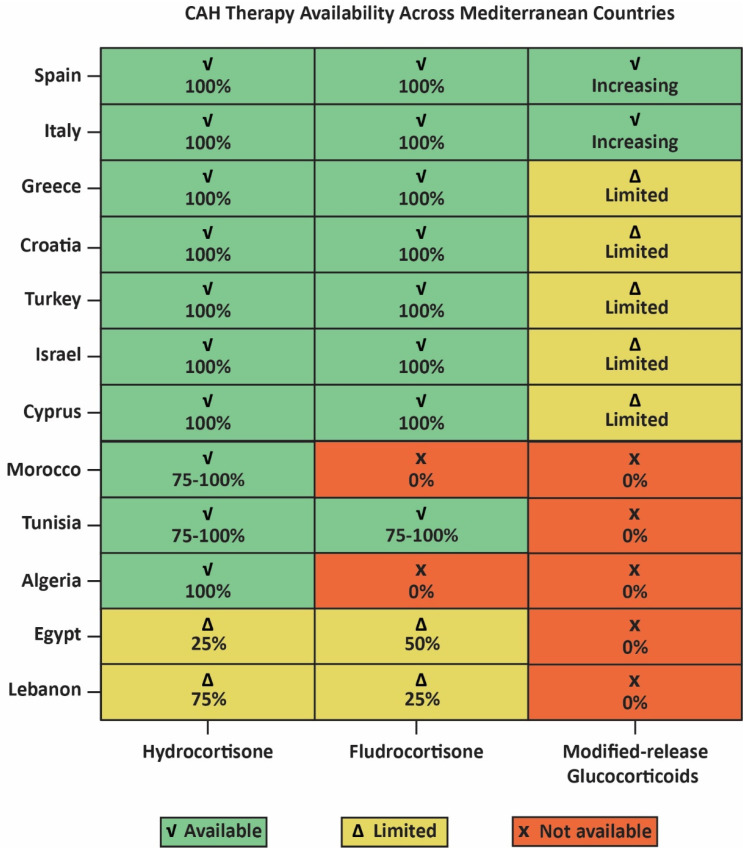
Regional differences in access to CAH therapies across Mediterranean countries. Hydrocortisone is widely available, whereas fludrocortisone and modern modified-release glucocorticoids show variable and often limited availability outside Southern Europe.

Data were collected from 23 Mediterranean and neighboring regions that included over 8.7 million screened newborns, genetic cohorts and clinically diagnosed individuals with CAH (Figure 1, Table 1). Most studies focused on 21-OHD (*CYP21A2* mutations), the predominant cause of CAH and included both classic (SW and SV forms) and NC-CAH forms. Diagnostic methods ranged from immunoassay-based 17-hydroxyprogesterone (17-OHP) measurement on dried blood spots (DBS) to second-tier liquid chromatography–tandem mass spectrometry (LC-MS/MS) and *CYP21A2* genotyping. The availability of replacement therapy with hydrocortisone and fludrocortisone varied substantially across regions (Figure 2) [22]. It should be noted that incidence estimates resulting from clinical cohorts in countries lacking established NBS may be subject to underreporting and selective case ascertainment, predominantly for the milder NC-CAH.

### 3.2. Genetic Structure and Founder Effects Across the Mediterranean

Genetic data across the Mediterranean Basin demonstrate considerable heterogeneity in the distribution of *CYP21A2* variants and denote differences in the population structure. Southern European populations generally present a wide-ranging allelic spectrum, consistent with historical admixture and comparatively low levels of consanguinity [10,23]. On the contrary, populations from the Eastern Mediterranean and the Balkans display evidence of recurrent mutations, including IVS2-13A/C>G and p.Pro30Leu, indicative of regional founder effects [24].

In numerous populations, higher carrier frequencies further support the role of allele enrichment. For instance, the high carrier frequency reported in Cyprus (~1:10) is suggestive of the impact of genetic drift and population isolation [23]. Analogous patterns have been identified in specific ethnic groups, such as Ashkenazi Jews, where NC-CAH associated variants occur at distinctly increased frequencies [11,25].

In North Africa, the joint effect of founder mutations and high consanguinity rates prospectively contributes to increased homozygosity and a higher frequency of classic CAH, although comprehensive population-based genetic data continue to be limited [26,27,28].

Summary of published newborn screening programs, clinical cohorts and genetic studies reporting incidence or prevalence of classic and non-classic CAH due to 21-OHD. The table includes sample size, diagnostic method (immunoassay, LC-MS/MS, or molecular), therapeutic drug availability and reported incidence or carrier frequencies by region. Data show marked regional variability, with higher incidence in North Africa and the Eastern Mediterranean and near-universal hydrocortisone but variable fludrocortisone access. (Abbreviations: dried blood spots (DBS); 21-hydroxylase deficiency (21-OHD); 17-hydroxyprogesterone (17-OHP); liquid chromatography–tandem mass spectrometry (LC-MS/MS); Multiplex Ligation-dependent Probe Amplification (MLPA).)

**Table 1:** Regional CAH incidence, prevalence, and therapy availability in Mediterranean countries (1986–2025).

Region/Period	Sample Size/ Study Type	CAH Type	Diagnostic Method	Therapy Availability	Incidence/Prevalence
Athens, Greece 2007–2009	45,000 (DBS program)	Classic	17-OHP immunoassay	Yes—Fludrocortisone & Hydrocortisone	2:45,000; 1:22,500 [29]
Catalonia, Spain 2017–2019	130,903 (DBS + hyperandrogenic women cohort)	Classic	17-OHP immunoassay	Yes—Fludrocortisone & Hydrocortisone	1:10,000–1:25,000 [30]
Spain 1990–2017	3,086,015 (DBS program)	Classic	17-OHP immunoassay	Yes—Fludrocortisone & Hydrocortisone	1:21,732
Italy (5 regions) 2006–2019	2,933,074 (DBS program)	Classic (SW & SV)	17-OHP + LC-MS/MS 2nd tier	Yes—Fludrocortisone & Hydrocortisone	1:17,699 [31]
Northeast Italy 2001–2021	862,521 (DBS program)	Classic	17-OHP (immunoassay, LC-MS/MS)	Yes—Fludrocortisone & Hydrocortisone	34 cases; 1:25,368 [32]
Northeast Italy 2001–2004	121,000 (DBS program)	Classic	17-OHP immunoassay	Yes—Fludrocortisone & Hydrocortisone	6 cases; 1:21,380 [33]
Italy Mainland & Sardinia 2018	293 (Clinical Cohort)	Classic & NC-CAH	*CYP21A2* sequencing/MLPA	Yes—Fludrocortisone & Hydrocortisone	NC-CAH: 1:1214 (Mainland), 1:2986 (Sardinia) [34]
Sicily, Palermo 2005–2016	141 women (Clinical Cohort)	NC-CAH	17-OHP + *CYP21A2* genotype	Yes—Fludrocortisone & Hydrocortisone	NC-CAH: 7% in hyperandrogenic women [35]
France (Including Southern part) 1996–2003	National (DBS program)	Classic	17-OHP immunoassay	Yes—Fludrocortisone & Hydrocortisone	1:15,000–1:16,000 [36]
Turkey 2017–2018	241,083 (DBS program)	Classic	17-OHP immunoassay + LC-MS/MS (some centers)	Yes—Fludrocortisone & Hydrocortisone	20 cases; ~1:12,000 [37]
Israel 1986–1992	113,846 (DBS program)	Classic	17-OHP immunoassay	Yes—Fludrocortisone & Hydrocortisone	National: 1:28,462; North Israel: 1:14,240 [38]
Cyprus 2006–2023	1222 (Clinical Cohort)	Classic & NC-CAH	CYP21A2 sequencing/MLPA/RFLP	Yes—Fludrocortisone & Hydrocortisone	18 cases, 1:38,650 [10]; carrier frequency 1:10 [39,40]
Croatia 1995–2006	532,942 (live births)	Classic	17-OHP + *CYP21A2* genotype	Yes—Fludrocortisone & Hydrocortisone	34 cases; 1:15,674 [41]
Slovenia	Clinical Cohort	Classic	17-OHP + *CYP21A2* genotype	Yes—Fludrocortisone & Hydrocortisone	No national incidence data; high frequency IVS2-13A/C>G and p.Pro30Leu [24]
Malta, Albania, Montenegro, Bosnia & Herzegovina	National Clinical Cohorts	Classic	17-OHP on DBS	Yes—Fludrocortisone & Hydrocortisone	No major national incidence data
Morocco 2013–2023	184 children (Clinical Cohort)	Classic	17-OHP + karyotype	Yes—Hydrocortisone: 75–100%; Fludrocortisone: 0%	1:5000–7000 [42,43]
Tunisia 2004, 2012	51 patients (Clinical Cohort)	Classic & NC-CAH	*CYP21A2* sequencing	Yes—75–100% both Fludrocortisone & Hydrocortisone	No major national incidence data [26]
Algeria ongoing since 1990	299 infants (Clinical Cohort)	Classic & NC-CAH	17-OHP immunoassay	Yes—Hydrocortisone 100%; Fludrocortisone 0%	No major national incidence data [44]
Egypt 2008–2009	7254 newborns (Clinical Cohort)	Classic & NC-CAH	17-OHP immunoassay	Yes—Hydrocortisone 25%; Fludrocortisone 50%	1:1209–1:1209 [27,28]
Lebanon 2000	25 patients (Clinical Cohort)	Classic & NC-CAH	17-OHP + *CYP21A2* sequencing/RFLP/Southern blot	Yes—Hydrocortisone 75%; Fludrocortisone 25%	No major national incidence data [45,46]

- Southern European Countries of the Mediterranean Basin

Across Southern European Mediterranean populations, newborn screening data designate that the incidence of classic CAH is relatively consistent, usually ranging from 1:15,000 to 1:25,000 live births [29,30]. Estimates derived from large-scale national and regional programs are found within this interval, with minor variability resulting from differences to screening algorithms and the employment of second-tier testing [31,32,33]. In contrast, NC-CAH demonstrates increased heterogeneity, with reported frequencies ranging from approximately 1:1200 to 1:3000, consistent with regional founder effect phenomena and population stratification [34,35].

b. Eastern Mediterranean and Balkans

In the Eastern Mediterranean and Balkan regions, the incidence of classic CAH shows wider variation, ranging approximately 1:12,000 to 1:36,000 [24,37,38,39,41]. Most population-based newborn screening studies correspond to an approximate incidence between 1:12,000 and 1:16,000, whereas higher estimates are characteristically consequent from smaller or genetically defined cohorts. Noticeable intra-regional heterogeneity is detected, likely as a result of differences in the genetic background, including consanguinity and founder mutations. Markedly, Cyprus exhibits a comparatively low incidence of classic CAH (~1:36,000) together with a high carrier frequency for NC-CAH (~10%), highlighting lack of agreement between disease incidence and carrier frequencies [10,39,40].

c. North Africa and the Middle East

Data from North Africa and the Middle East represent a higher and more variable burden of CAH. Reported incidences of classic CAH are characteristically in the range of 1:5000 to 1:7000, although considerably higher estimates have been reported in small pilot screening studies and should be anticipated with caution as a result of methodological limitations [26,27,28,42,43,44]. The absence of extensive national newborn screening programs and varied access to glucocorticoid and mineralocorticoid therapy further limit the comparability of epidemiological data across this region [45,46].

Overall, these findings propose a geographical gradient in CAH incidence across the Mediterranean Basin, with relatively steady estimates in Southern Europe, increased variability in the Eastern Mediterranean and Balkans, and higher reported incidence in North Africa and the Middle East. This variation likely involves a combination of genetic factors, screening methods and healthcare system differences rather than true epidemiological deviation alone.

### 3.3. Glucocorticoid Prescribing and Treatment Patterns

Across the Mediterranean Basin, the management of CAH, particularly due to 21-OH deficiency, relies on lifetime glucocorticoid replacement therapy, always combined with mineralocorticoid supplementation (fludrocortisone) in the salt-wasting forms (Table 1). Regardless of common therapeutic approaches, substantial regional differences exist in drug availability, prescribing trends and availability to modern formulations (Figure 2, Table 2).

Historically, hydrocortisone has been the basis of CAH treatment and until today remains the first-line therapy in most countries, especially in infants and growing children and adolescents to replace inadequate cortisol synthesis and suppress excess adrenal androgen production via negative feedback on the hypothalamic–pituitary–adrenal (HPA) axis [2]. Hydrocortisone is preferred because of its short half-life and its ability to more closely mimic physiological cortisol replacement while lessening adverse effects on growth. Registry data from international cohorts such as the I-CAH registry indicate that hydrocortisone continues to be the preferred medication for the majority of glucocorticoid prescriptions worldwide [47]. In addition to hydrocortisone, several other longer-acting glucocorticoids (Prednisolone, Dexamethasone) have traditionally been used, particularly in adolescents and adults [48]. These medications induce stronger suppression of ACTH and therefore lessen the adrenal androgen production but are linked with increased perils of metabolic complications and should be used with caution.

During the past decade, significant pharmacologic research led to the development of modern modified-release glucocorticoids designed to improve the physiological circadian rhythm of cortisol secretion. These include Alkindi, hydrocortisone granules that permit precise dosing in infants and young children; Efmody, a modified-release hydrocortisone formulation that provides delayed nocturnal cortisol delivery; Plenadren, a dual-release hydrocortisone preparation that enables once-daily dosing; and Acecort, another improved hydrocortisone preparation that is currently under evaluation [49]. These newer formulations are designed to better mimic the circadian secretion of cortisol, improve metabolic consequences, reduce androgen excess and make patients more compliant to the treatment.

Data from the recent international study “Glucocorticoid Prescribing Trends in Congenital Adrenal Hyperplasia (2017–2023)”, which was based on the International-Congenital Adrenal Hyperplasia (I-CAH) registry (https://sdmregistries.org/i-cah/, accessed on 12 March 2026), demonstrated that although hydrocortisone remains the principal therapy, usage of contemporary glucocorticoids is steadily increasing [50]. However, their adoption remains rather limited, with only a small percentage of approximately 5% of patients transitioning from traditional therapies to these newer therapies. By 2023, availability across the 20 participating centers of the above study had increased substantially, with Alkindi accessible in approximately 54% of centers, Efmody in 46%, Plenadren in 33% and Acecort in 15%. Currently, these medicines are primarily used in high-income European healthcare systems, where regulatory approval and compensation policies support patient access, while access in North Africa and parts of the Eastern Mediterranean remains limited due to regulatory barriers and cost limitations.

**Figure 3:**
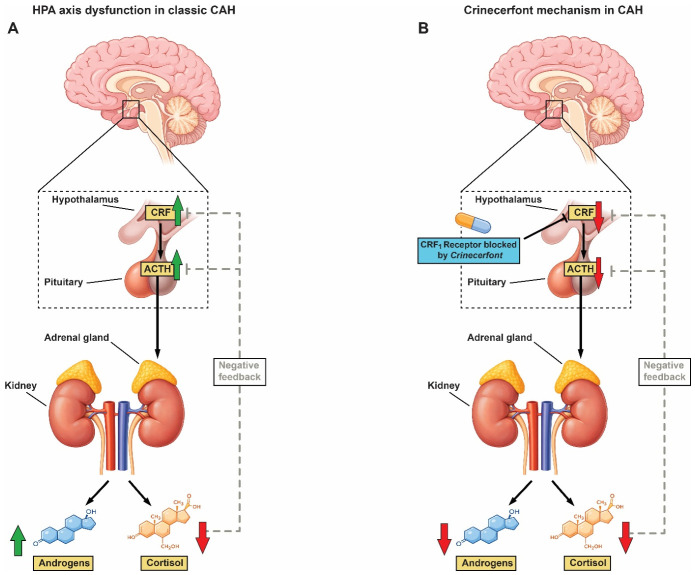
HPA axis dysregulation in classic CAH and mechanism of action of Crinecerfont. (**A**) In classic CAH, impaired cortisol synthesis results in reduced negative feedback at the hypothalamus and pituitary, leading to increased corticotropin-releasing factor (CRF) and adrenocorticotropic hormone (ACTH) secretion and consequent adrenal androgen excess. (**B**) Crinecerfont, a corticotropin-releasing hormone receptor 1 antagonist, inhibits CRH signaling at the pituitary, leading to reduced ACTH secretion and decreased adrenal androgen production. Cortisol levels remain low and negative feedback remains impaired.

Currently, the most recent drug for the treatment of classic CAH is Crinecerfont, which received its first approval by the U.S. Food and Drug Administration [51]. Regulatory approval globally by other countries and the European Medicines Agency (EMA) is at present under evaluation. The drug is a non-steroidal corticotropin-releasing factor type 1 (CRF1) receptor antagonist that suppresses excessive ACTH secretion and was approved as an adjunct to glucocorticoid therapy for adults and children aged ≥4 years with classic CAH. Clinical trials demonstrated that treatment with Crinecerfont significantly reduced ACTH and androgen levels and enabled reductions in supraphysiologic glucocorticoid doses, potentially decreasing long-term steroid-related adverse effects (Figure 3) [52].

In Southern European Mediterranean countries such as Italy, Spain, France and Greece, CAH treatment usually follows international endocrine guidelines, with hydrocortisone as the standard therapy in children and fludrocortisone used in salt-wasting disease. In specialized endocrine centers, newer medications such as Alkindi or Efmody may gradually be available. In Eastern Mediterranean and Balkan countries (e.g., Turkey, Israel, Cyprus), treatment also relies principally on hydrocortisone, while access to modern formulations is more limited and generally restricted to tertiary care centers.

In contrast, North African and some Middle Eastern countries encounter greater challenges regarding medication accessibility. Clinical reports from Morocco, Algeria, Tunisia, Egypt and Lebanon specify that hydrocortisone is commonly used and often the only glucocorticoid available, while fludrocortisone availability is negligible or absent. Modern glucocorticoid formulations are generally not commonly available as a result of economic and governing barriers.

Overall, the present therapeutic procedures across the Mediterranean Basin resembles to a transition phase, in which traditional hydrocortisone therapy remains the standard treatment, while new modified-release glucocorticoids are slowly established in particular healthcare systems. Persistent differences in drug availability, screening programs and endocrine care set-up continue to impact treatment approaches and consequences in patients with CAH.

## 4. Discussion

This systematic review provides an updated synopsis of the epidemiology, diagnostic practices and management of CAH across countries of the Mediterranean Basin. Analysis of 28 studies that included newborn screening programs, genetic cohorts and clinical series identified noteworthy regional heterogeneity in disease incidence, diagnostic set-up and endocrine care provision.

The global incidence of classic CAH due to 21-OHD generally ranges between 1:10,000 and 1:25,000 live births and the epidemiological findings in regions with established newborn screening programs, such as Spain, Italy, France and Greece were also found to be the case. These estimates are consistent with previously reported global incidence ranges for classic CAH [1,5,12,36]. However, noticeably higher incidence estimates were detected in some countries of the Eastern Mediterranean and North Africa, including Morocco and Egypt [27,28,39,42,43]. This geographic variation is illustrated in Figure 1. These higher estimates may somewhat be the consequence of high consanguinity rates and strong founder effects affecting the *CYP21A2* gene, which have been documented in several Mediterranean populations. Genetic studies included in this review further support the existence of regional founder effects that have an impact on the distribution of CAH. For example, the unusually high carrier frequency of 1:10 that is reported in Cyprus highpoints the impact of historical population structure and genetic drift on disease prevalence [10,23]. On the other hand, the absence of universal newborn screening (NBS) in some countries may lead to delayed clinical diagnosis and potential overrepresentation of severe CAH cases. Similar patterns of mutation clustering have also been observed in other Mediterranean populations, including Italy, Slovenia and Israel [24,31,34,35,38]. These findings underline the importance of molecular diagnostics that play in the prediction of disease severity, as genotype–phenotype correlations contribute to the guiding of the appropriate therapeutic strategies. Additionally, they are consistent with established population genetic models, demonstrating that consanguinity increases autozygosity and autosomal recessive disorders, while founder mutations contribute to regional clustering of pathogenic variants [53,54].

Diagnostic methodologies across the Mediterranean Basin also reveal noteworthy discrepancies. In countries with well-established NBS programs, such as Italy, Spain, and Turkey, measurement of 17-OHP from dried blood spots is currently the main screening method, often accompanied by second-tier testing using liquid chromatography–tandem mass spectrometry to improve specificity. On the contrary, some countries in North Africa and the Eastern Mediterranean rely primarily on clinical diagnosis and targeted biochemical testing due to the absence of NBS programs (Table 1). This discrepancy leads to delayed diagnosis and could increase the risk of adrenal crises in affected infants.

The therapeutic approaches for CAH across the Mediterranean Basin are principally in accordance with the international endocrine guidelines (Table 2). Lifetime glucocorticoid replacement therapy, most commonly with hydrocortisone, remains the basis of treatment for both classic and non-classic CAH [2,7,20]. Hydrocortisone is favored in pediatric populations for its brief half-life and more physiological cortisol replacement profile, whereas longer-acting glucocorticoids may well be used in adolescents and adults to increase the suppression of ACTH and adrenal androgen production [55,56]. Nonetheless, significant regional differences in medication availability were recognized. As depicted in Table 1, hydrocortisone therapy seems the preferable choice across European Mediterranean countries, while fludrocortisone availability remains variable in several North African countries, conceivably limiting optimum management of salt-wasting CAH.

Over the past decade, pharmacological developments have presented modified-release hydrocortisone formulations designed to improve the physiological circadian rhythm of cortisol secretion. These formulations, including Alkindi, Efmody and Plenadren, have improved the metabolic outcomes and treatment competence. Nevertheless, their availability is principally restricted to higher-income healthcare systems, and their usage in Mediterranean countries outside Western Europe is at this time limited [50,57,58].

More lately, novel non-steroidal therapeutic approaches have arisen. The selective corticotropin-releasing factor type 1 receptor antagonist Crinecerfont represents the most recent pharmacological advancement in CAH management [51,58,59]. Following its first regulatory approval by the U.S. Food and Drug Administration, clinical trials have established that Crinecerfont meaningfully diminishes ACTH and adrenal androgen levels and permits reduction in supraphysiologic glucocorticoid doses in patients with classic CAH (Figure 3) [51]. Even though regulatory evaluation in other world regions, as well as by the European Medicines Agency (EMA), is ongoing, this therapeutic method may well represent a significant future strategy to decrease long-term complications connected with chronic glucocorticoid exposure.

Regardless of these developments, several challenges persist in the Mediterranean region. Partial pharmacogenomic infrastructure, absence of national disease registries and inadequate access to endocrine medications continue to delay the implementation of personalized medicine approaches. Additionally, the absence of standardized regional NBS policies complicates precise epidemiological surveillance and delays early detection in some countries.

Regardless of the comprehensive scope of this overview, there are some limitations that should be noted. First, the included studies display extensive heterogeneity in design about NBS programs, retrospective clinical series and genetic cohort analyses, which may limit comparability across regions. Second, the absence of uniform diagnostic criteria and variability in biochemical and molecular testing methods across countries may have created discrepancies in reported incidence and phenotype classification. Third, notable regional data gaps exist, mainly in parts of North Africa and the Eastern Mediterranean, where inadequate availability of national registries may distort the accuracy of epidemiological estimates. These limitations stress the need for coherent methodologies and improved data collection systems to allow more robust regional evaluations in future research.

Overall, the findings of this review determine the multifaceted interaction between genetic background, healthcare infrastructure and public health policies in determining the epidemiology and management of CAH across the Mediterranean Basin. Addressing these discrepancies will necessitate coordinated regional efforts intending to expand NBS programs, establish molecular diagnostic infrastructures and improve access to crucial endocrine therapies.

## Figures and Tables

**Table 2:** Modern glucocorticoid availability for CAH treatment in Mediterranean countries that aim to improve physiologic cortisol replacement and treatment adherence.

Region	Country	Modern Glucocorticoids (Alkindi, Efmody, Plenadren, Acecort)
Southern Europe	Greece	Limited/specialized centers
	Italy	Increasing availability
	Spain	Increasing availability
	France	Available in specialized centers
Eastern Mediterranean	Turkey	Limited
	Israel	Some access
	Cyprus	Limited
Balkans	Croatia	Limited
	Slovenia	Limited
North Africa	Morocco	Not widely available
	Tunisia	Not widely available
	Algeria	Not available
Middle East	Egypt	Not available
	Lebanon	Very limited

## Data Availability

No new data were created or analyzed in this study. Data sharing is not applicable.

## Abbreviations

CAH	Congenital Adrenal Hyperplasia
21-OHD	21-hydroxylase deficiency
21-OH	21-hydroxylase enzyme
*CYP11B1*	11β-hydroxylase deficiency
*HSD3B2*	3β-hydroxysteroid dehydrogenase deficiency
*CYP17A1*	17α-hydroxylase/17,20-lyase deficiency
*STAR*	Steroidogenic acute regulatory protein deficiency
*CYP11A1*	Cholesterol side-chain cleavage enzyme deficiency
*POR*	P450 oxidoreductase deficiency
ACTH	Adrenocorticotropic hormone
SW	Salt-wasting
SV	Simple-virilizing
ATF	Article Type Filter
17-OHP	17-hydroxyprogesterone
NC-CAH	Non-Classic Congenital Adrenal Hyperplasia
DBS	Dried blood spots
LC-MS/MS	Liquid chromatography–tandem mass spectrometry
MLPA	Multiplex Ligation-dependent Probe Amplification
HPA	Hypothalamic–Pituitary–Adrenal
I-CAH	International-Congenital Adrenal Hyperplasia
EMA	European Medicines Agency
CRF1	Corticotropin-releasing factor type 1
NBS	Newborn Screening
